# Associations between Patient-Reported and Clinician-Reported Outcome Measures in Patients after Traumatic Injuries of the Lower Limb

**DOI:** 10.3390/ijerph19053140

**Published:** 2022-03-07

**Authors:** Walter Bily, Jakob Jauker, Helena Nics, Vincent Grote, Michael Pirchl, Michael J. Fischer

**Affiliations:** 1 Department of Physical Medicine and Rehabilitation, Ottakring Clinic, Vienna Health Association, A-1160 Vienna, Austria; walter.bily@gesundheitsverbund.at (W.B.); jakob.jauker@gesundheitsverbund.at (J.J.); 2 Department of Physical Medicine and Rehabilitation, Floridsdorf Clinic, Vienna Health Association, A-1210 Vienna, Austria; helena.nics@gesundheitsverbund.at; 3 Ludwig Boltzmann Institute for Rehabilitation Research, A-1140 Vienna, Austria; michael.pirchl@rehabilitation.lbg.ac.at (M.P.); michael.fischer@reha-kitz.at (M.J.F.); 4 Department of Physical Medicine, Rehabilitation and Occupational Medicine, Medical University of Vienna, A-1090 Vienna, Austria; 5 Vamed Rehabilitation Center Kitzbühel, A-6370 Kitzbühel, Austria

**Keywords:** traumatic lower limb injury, rehabilitation, patient-reported outcome measures, clinical outcome assessments

## Abstract

Both clinician-reported outcome measures (CROMs) measures and patient-reported outcome measures (PROMs) are applied to evaluate outcomes in rehabilitation settings. The previous data show only a low to moderate correlation between these measures. Relationships between functional performance measures (Clinician-Reported Outcome Measures, CROMs) and Patient-Reported Outcome Measures (PROMs) were analysed in rehabilitation patients with traumatic injuries of the lower limb. A cohort of 315 patients with 3 subgroups (127 hip, 101 knee and 87 ankle region) was analysed before and after 3 weeks of inpatient rehabilitation. All three groups showed significant improvements in PROMs with low to moderate effect sizes. Moderate to high effect sizes were found for CROMs. Correlation coefficients between CROMs and PROMs were low to moderate. The performance consistency between PROMs and CROMs ranged from 56.7% to 64.1%. In this cohort of rehabilitation patients with traumatic injuries, CROMs showed higher effect sizes than PROMs. When used in combination, patient-reported outcome and performance measures contribute to collecting complementary information, enabling the practitioner to make a more accurate clinical evaluation of the patient’s condition.

## 1. Introduction

Ultimately, the aim of postoperative rehabilitation is to enable the patient to fully perform daily, leisure and sports activities and to maintain their ability to work. Rehabilitation after lower limb fractures supports pain control and restores strength, range of motion and function, helping the patients reintegrate into their normal life and work schedules.

In order to monitor therapy effects and the outcomes of postoperative rehabilitation, different clinician-reported outcome measures (CROMs) and patient-reported outcome measures (PROMs) are commonly used. Various studies have been carried out to analyse postoperative recovery with either CROMs or PROMs, enabling clinicians to evaluate the effects of therapeutic interventions, identify factors that influence the outcome and improve their performance constantly [1,2]. An analysis of previous studies comparing PROMs and CROMs indicates that a moderate correlation between the two exists, showing that self-reported and performance measures provide different kinds of clinical information about the functional state of the patient [3,4,5]. Stamm et al. [6] stated that comparing PROMs and CROMs is necessary to analyse the aspects of the patients’ functional status that are covered by each instrument and determine whether it is possible to predict performance measures by examining patient-reported outcomes and vice versa.

PROMs are standardised, validated questionnaires that have been designed to measure the progress with focus of a patients’ perspective. Their sensitivity to the smallest detectable changes makes it possible to determine and statistically analyse the effectiveness of a rehabilitation programme [1,2,7]. While CROMs measure specific functions or abilities that enable the clinician to obtain an outcome for a standardised measurement of the patients’ performance in a clinical setting, they do not allow them to evaluate the performance in daily activities [8,9,10,11]. PROMs, on the other hand, can provide information about the patients’ quality of life and performance in a daily living setting. If both aspects are considered, a more holistic approach can be taken toward the assessment and evaluation of a patient’s physical condition. This means that PROMs serve as an important tool that clinicians and physiotherapists can use to evaluate the patient’s self-reported current state and progress during the rehabilitation process. Measuring CROMs and PROMs systematically during rehabilitation also allows the clinician to evaluate how treatment or time effects alter a patient’s performance status [10,11]. As stated in Ashford et al. [1], PROMs and CROMs can be viewed as complementary methods. Both approaches are used to evaluate clinical outcomes and the effectiveness of interventions, each with different strength and limitations. PROMS are gaining importance as efforts to promote patient-centred research [12]. Lower confidence in “subjective” PROMs compared with “objective” clinical measures is not eligible [13]. They give insight into potential psychosocial factors and patients’ subjective perception like their expectations, values, feelings, own functioning and proprioception [14].

The main objectives of this study were to evaluate the effect sizes of changes, correlations and consistencies between PROMs and CROMs in rehabilitation patients after lower limb injuries before and 3 weeks after inpatient rehabilitation. To assess a patient’s functional and health status before and after rehabilitation, appropriate PROMs (Numeric Pain Rating Scale [NPRS], HAQ-DI, WOMAC (Western Ontario and McMaster Universities Osteoarthritis Index) and EQ-5D-5L) [9,15,16,17] and CROMs (TUG and aROM) [18,19] were administered (see Section 2.2). While TUG can be used to assess the mobility and risk of falls, a ROM measurement can be used to evaluate the actual functional status of the specific joints. The NPRS reflects the pain, while the HAQ-DI represents the health status and patient-oriented outcomes. In addition, the EQ-5D-5L reveals the quality of life, and the WOMAC assesses the patient’s subjective perception of their functional limitations.

We hypothesised that we would find moderate correlations between specific PROMs and CROMs in patients with posttraumatic lower limb limitations, as the TUG and the aROM are used to measure mobility and functional status. For this reason, our assumption was that they could be applied to reflect the actual functional status embedded in a daily living setting. We only found limited data about the context between PROMs and CROMs. Our intent was to contribute to a better understanding of the relationships between clinician-reported measurements and patient-reported data and identify discrepancies between these measurements.

This study is part of a retrospective data analysis project on “clinician and patient-reported outcome measures” before and three weeks after a rehabilitation programme. Part of the dataset was published elsewhere [20]. In the present paper, data from traumatic limb injury patients were analysed.

## 2. Materials and Methods

### 2.1. Study Design and Participants

A sample of 315 patients (120 men, 195 women, age = 59.8 ± 15.2 years) who experienced traumatic fractures of the lower limb was selected out of a total of 5495 patients who had undergone orthopaedic inpatient rehabilitation between January 2018 and March 2020.

**Figure 1:**
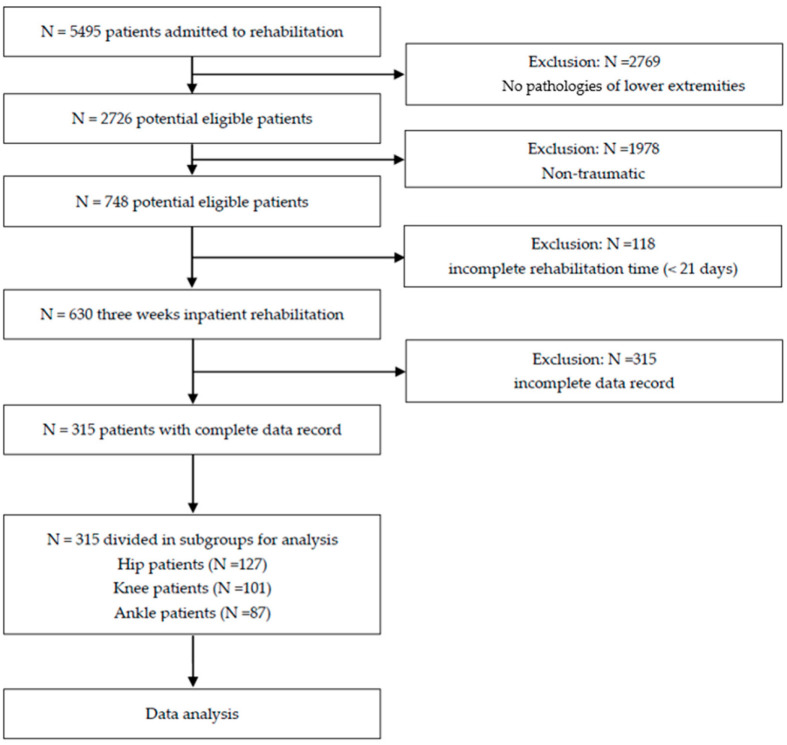
Flow diagram of study participants.

Patients were included in the study if they had received a fracture diagnosis of the lower limb, actively participated in three weeks of inpatient rehabilitation and had a complete data record for all CROMs and PROMs. Orthopaedic patients with non-traumatic diagnoses were excluded (Figure 1).

The rehabilitation programme lasted on average 2–3 h per day and consisted of daily personalised physiotherapy, including exercise therapy, electrotherapy and hydrotherapy treatments, lymphatic drainage and massage as well as hydrotherapy. These treatments amounted to at least 1800 therapy minutes during the three-week rehabilitation programme. The total average individual physiotherapy time was comparable in all groups, ranging between 471 and 496 therapy minutes.

Selected patients were assigned to one of three groups: the hip, knee or ankle group. The demographic characteristics of the subjects are reported in Table 1.

The informed consents of all patients were provided at the beginning of the rehabilitation process. The clinical study received approval for the study from the Ethics Committee of the Medical University of Innsbruck, Austria (1158/2019), which was entered retrospectively on 14 August 2020, into the German Register for Clinical Studies (trial registration number: DRKS00022854).

### 2.2. Outcome Measures

CROMs (aROM and TUG) and PROMs (WOMAC, NPRS, HAQ-DI and EQ-5D-5L) were measured before and three weeks after inpatient rehabilitation.

The NPRS is a segmented numeric version of the visual analogue scale (VAS), ranging from zero to ten, with these numbers indicating a state from no pain and the worst pain. The patient selects a whole number (integers of 0–10) that best reflects the intensity of their pain. The NPRS serves as a reliable and valid measure of pain intensity due to the ease of its use and high level of responsiveness [15]. Compared to the VAS and verbal rating scale (VRS), the NPRS has proved to be superior in terms of its practicability and sensitivity to changes in pain [21].

The HAQ-DI is a PROM that is used to assess a patient’s restrictions in terms of their activities of daily living; it was originally developed to assess patients with rheumatoid arthritis. The commonly used version includes 20 questions scored from 0 to 3, with these numbers corresponding to “without any difficulty”, “with some difficulty”, “with much difficulty” and “unable to do without help”. The resulting average score falls within a range of 0 to 3 and is influenced by pain, psychological factors and health conditions. As noted by Bruce et al. [16], the HAQ-DI has been repeatedly validated as a reliable measurement.

The EQ-5D-5L questionnaire is used to assess quality of life based on the health status. This generic measure reflects five dimensions of daily living (mobility, self-care, usual activities, pain/discomfort and anxiety/depression) and five levels of severity ranging from 1 = no problems to 5 = extreme problems. Furthermore, the result is indexed by applying an algorithm, which yields a number ranging from 0 to 1 that expresses the current health status. The EQ-5D-5L has been proven to be a valid and reliable tool in group comparisons, is responsive to change and is easy to use [17].

The WOMAC is a questionnaire with 24 items comprising 5 for pain, 2 for stiffness and 17 for functional limitations. The response levels for each question range from 0 (i.e., best) to 10 (i.e., worst). Dividing the result by 24 allows the user to perform statistical calculations with relative values. As a self-reported measure, the WOMAC is considered a reliable and valid tool that can be used to assess the satisfaction of osteoarthritis patients after undergoing hip or knee arthroplasty [9].

The TUG is applied to assess the general mobility and risk of falls in patients. The patients in this study used their usual footwear and did not use assistive walking devices; they were instructed to rise from a standard armchair using the armrests, walk a distance of three meters, turn around, walk back and sit down again as quickly as possible. The time that elapsed from the point that the patient lost contact with the chair’s backrest to the point they resumed their sitting position was recorded with a standard stopwatch (precision: ±0.1 s). The TUG is a reliable and valid tool to quantify the functional ability of a patient [19]. Referring to Kennedy et al. [22], the reliability of the TUG meets the standards for group application and is preferred in the acute postoperative phase. The reliability of the TUG has been reported to range from 0.95 to 0.97 ICC in comparable settings/patient populations [23].

Each joint has a specific range of motion that can be measured in degrees using a goniometer. If reference values are available, it is possible to report aROM as a percentage of normal values which is preferable for interpreting short-term results. To examine aROM, the subject moves the joint by performing muscle contractions without the assistance of the examiner [18]. The high reliability of the goniometer with regard to measuring active ROM has been demonstrated in previous studies [24].

### 2.3. Statistical Analysis

Descriptive statistics were given as a mean ± SD. Individual missing values in 5–10% of cases were not replaced for statistical analysis (not used for calculations). As assumptions for parametric testing could not be met, the Wilcoxon signed-rank test was performed. The Holm–Bonferroni correction for multiple comparisons was used to reduce the type 1 error rate. The effect sizes were interpreted according to Cohen, classifying > 0.80 as strong, 0.50–0.79 as moderate and 0.20–0.49 as weak. The correlation between objective measures and PROMs was interpreted for both t1 and t2 scores using Spearman’s rank correlation coefficients.

To reduce a potential ceiling effect for patients who exhibited good performance at the beginning of the rehabilitation and a reduced potential for further improvement, a method mentioned earlier [20] was used to assess their performance considering their initial functional capability. Applying the formula t2 + Δ reproduces the performance as well as the functional capability and minimises the potential ceiling effect. By transforming the performance scores into normal scores by calculating rankits and using appropriate formula, it was possible to achieve better interpretability.

The differences between the normal scores of objective measures and the PROMs were calculated to show the level of consistency. Subsequently, three groups were defined to present the differences as a percentage of patients who had normal scores in both measures within 1 SD of each other (high consistency), between 1 and 2 SDs (average) or over 2 SDs (low).

To perform statistical analyses, SPSS Statistics for Windows, Version 25.0 (IBM Inc., Armonk, NY, USA) was used.

## 3. Results

### 3.1. Baseline Values and Effect Sizes of Change

The sample, which included 127 hip patients, 101 knee patients and 87 ankle patients, was analysed. PROMs and CROMs at baseline and changes as well as the Cohen’s *d_z_* values for all three groups are given in Table 2. Patients in the hip group were explicitly older than knee and ankle patients.

After three weeks of intervention, all groups showed significant improvements in PROMs, concerning the HAQ-DI (*p* < 0.05), NPRS (*p* < 0.05–0.001) and WOMAC score (*p* < 0.001). Similarly marked improvements were shown for pain and health subscales (EQ-5D-5L), but only in knee and ankle patients (*p* < 0.01–0.001).

Effect sizes were low for HAQ-DI in all groups and NPRS in the hip group, and moderate effect sizes were seen for NPRS in the knee and ankle groups (Cohen’s *d_z_* 0.53–0.54). For EQ-5D-5L, low effect sizes were seen in the ankle and knee groups and for health and pain subscales and for usual activities subscale (0.35–0.44, 0.20–0.36, Table 2). The WOMAC score showed moderate effects (0.52–0.56) in all three groups.

Highly significant improvements were found for aROM in all three groups and for TUG in the hip and knee groups (*p* < 0.001) with high effect sizes for aROM (Cohen’s *d_z_* 0.88–1.06) and moderate to high effect sizes for TUG (Cohen’s *d_z_* 0.41–0.89, Table 2).

### 3.2. Correlations

Concerning the correlation between CROMs and PROMs before (t1) and after intervention (t2), the Spearman’s correlation coefficient shows high effect sizes for TUG and HAQ-DI in all groups (0.57–0.71) as well as for aROM and HAQ-DI for the hip and knee groups at t1 (0.53–0.55, Table 3). Low to moderate correlations were found between CROMs and EQ-5D-5L, as well as between NPRS and WOMAC. The average correlation values [25] show moderate effects between TUG and PROMs in the hip and ankle groups, and lower effects were seen between aROM and PROMs in the hip and ankle groups. Only small differences in the average correlations were observed between CROMs and PROMs in the knee group.

### 3.3. Consistency of Performance

A high consistency of performance between subjective PROMs and objective measurements was found in 56.7–61.3% of the patients for aROM and 57.2–64.1% for TUG, while 8.9–14% of the patients showed low consistency scores between PROMs and CROMs (Table 4).

**Figure 2:**
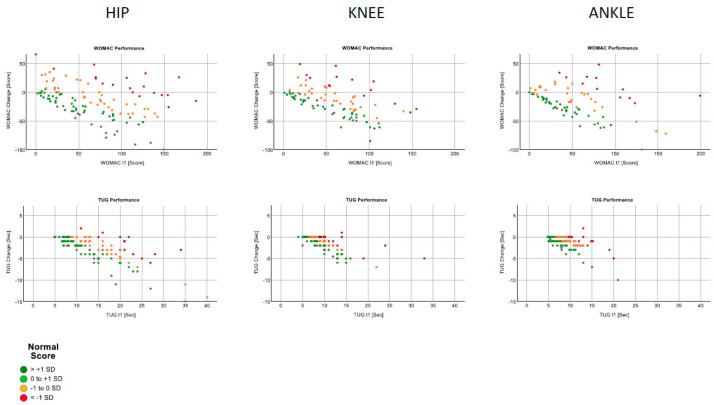
TUG and WOMAC improvements vs. performance classifications.

The behaviours of the WOMAC and TUG at the beginning of rehabilitation and in relation to the change observed are shown in Figure 2.

In general, the values improved over the course of the rehabilitation. Two effects were observed. First, the differences between the groups were less pronounced; the correlations were similar, and a stronger relationship was seen between the baseline values and their changes in the TUG than in the WOMAC scores. Second, the hip subgroup with older patients was the most heterogeneous.

## 4. Discussion

Our goals were to analyse the relations between particular PROMs and CROMs in patients who had experienced injuries of the lower limb with respect to effect sizes, correlations and consistency during a three-week rehabilitation process. We expected to see high effect sizes of change, moderate correlations and reliable consistencies of performance between these PROMs and CROMs.

After three weeks of inpatient rehabilitation, patients in all groups (hip, knee and ankle region) showed marked improvements in PROMs (HAQ-DI, NPRS and EQ-5D-5L), with low (EQ 5D-5L and HAQ-DI) to moderate effect sizes (NPRS and WOMAC).

The assessment of the CROMs revealed significant improvements with high effect sizes in aROM and moderate to high effect sizes in TUG after the intervention phase (Table 2).

Both PROMs and CROMs were sensitive to changes in the subjective and objective measurements. The effect sizes of objective performance measures were clearly higher for CROMs [20].

We did not find a tendency to overestimate self-reported measures in our late postoperative sample, contrary to the results reported for another sample of early postoperative total knee arthroplasty (TKA) patients [26].

In our setting, PROMs could be less sensitive to changes in this particular patient sample and late rehabilitation phase, considering the fact that self-reported and performance measures have different sensitivities in the early and late rehabilitation phases [3]. Clinicians must consider that PROMs could also be affected by other factors, such as the patients’ expectations, educational level, comorbidities and body mass index (BMI) [27,28]. The significance of each outcome measure (both PROMs and CROMs) needs to be assessed critically and using sophisticated methods, as well as always with respect to the particular patient sample and the actual time slot.

We found strong correlations between TUG and HAQ-DI in all groups and between aROM and HAQ-DI in the hip and knee groups [20]. The results of the dataset analysis indicate that the TUG showed stronger associations with PROMs than with aROM, including more functional performance components and reflecting functional strength, balance and mobility. The persistent/continuous high correlation observed between TUG and HAQ-DI could be partially explained, because both tools are used to evaluate functional domains of activities of daily living. All other parameters only showed low to moderate correlations between PROMs and CROMs, as has been reported in previous studies [4,29,30]. In contrast consistency of performance for t2 + Δ showed good agreement between PROMs and CROMs for aROM and TUG in more than half of the patients, while up to 14% of the data showed a low consistency between PROMs and CROMs (Table 4).

In a clinical setting, PROMs or CROMs are usually measured at a specific, assigned time or after a specific period has elapsed and are used to report the actual state of the patient at one specific moment or to detect changes over time. Measuring CROMs and PROMs regularly during the rehabilitation allows the clinician to evaluate changes in the patients’ performance status that are caused by treatment or time effects [3].

If we consider that PROMs and CROMs show different patterns of progress, practitioners can refer to recovery curves to choose appropriate measures and time points for evaluation [1,3]. Directly comparing the self-reported measures with performance measures of physical function after a specific period—while considering the fact that peaks of the growth curves might not be met—enables practitioners to further analyse recovery in rehabilitation patients [3].

The responsiveness of the measures varies according to the time interval after the injury or surgery. Patients after TKA were reported as needing two to four months to achieve a physical functional status comparable to their status prior to the injury. PROMs were superior in terms of representing early improvements, while CROMs were superior two to four months after TKA [26,31]. In a sample of hip and knee arthroplasty patients, Stratford et al. [30] showed different patterns of recovery curves between PROMs and CROMs. Values for PROMs (WOMAC PF, lower extremity function scale) improved much earlier and already reached preoperative values 2–3 weeks after operation, but the values from CROMs required 6–8 weeks (TUG, 6MWT) to recover to those prior to the operation.

While questionnaires and scores are cost-efficient and easy to apply, they are subjective because they include self-reported data. This means that they are influenced by the process of self-assessment and the patients’ subjective perceptions and expectations. Provided that they are applied in standardised and correct settings, PROMs can deliver reliable and consistent data, especially when taken at the mid- and long-term follow-up examination after TKA [32]. When they are interpreted in a critical appraisal, PROMs represent a practical and valuable tool in patient evaluation. We agree that the isolated use of PROMs alone cannot be recommended because they partially reflect the patients’ subjective impressions, and patients tend to overestimate their recovery and abilities, especially in early rehabilitation phases [30].

Clinician and patient-reported outcome measures of physical function appear to assess partially different but overlapping aspects of a patients’ abilities and impairments.

In our sample of posttraumatic rehabilitation patients, CROMs showed higher effect sizes than PROMs. The best correlations were found between CROMs and HAQ-DI.

When used in combination, patient-reported and performance measures contribute to the collection of complementary information, enabling the practitioner to make a more accurate clinical evaluation of the patients’ conditions.

Due to the use of standardised performance profiles, external reviews and the fact that insurers centrally control the assignment of modalities, we assume that the initial values and outcomes are representative for inpatient orthopaedic rehabilitation patients after traumatic injuries of the lower limb in Austria. 

The main limiting factors in our study were the retrospective cohort design and a naturally inhomogeneous posttraumatic patient sample. Both of these limitations, however, are offset by the use of adequate sample sizes.

Our results should be assessed considering the fact that our groups were not stratified by BMI and gender, aspects that can affect the results of outcome measures. Moreover, our data could have been influenced by the relatively short observation duration; longer surveillance periods could produce different results, especially because the peak of rehabilitation effects might not have been reached within this short period.

## 5. Conclusions

Our results indicate that PROMs and CROMs are reliable assessment tools in short-term rehabilitation programmes. While the levels of consistency were similar for both measures, CROMs showed higher effect sizes than PROMs in this sample of posttraumatic rehabilitation patients. A goal-oriented choice of patient-reported and clinical outcome measurement tools is required to obtain a comprehensive picture of a patient’s abilities or deficits. The study findings underline the importance of taking both PROMs and CROMs to get a multidimensional view and best medical outcome quality. Our study provides a valid basis for other researchers or healthcare professionals who commonly assess the quality of medical outcomes based on routine data to detect conflicting results and non-responders, and to support further research on possible critical or moderating success factors.

## Figures and Tables

**Table 1:** Demographic data for subjects in all three groups.

	Hip	Knee	Ankle
*n* (m/f)	127 (49/78)	101 (38/63)	87 (33/54)
Age	68.9 ± 13.1	54.2 ± 13.6	53.2 ± 13.4
Individual therapy minutes	471.5 ± 98.0	483.6 ± 118.6	495.9 ± 124.0

Mean ± SD.

**Table 2:** PROMs and CROMs at baseline (t1), changes (t2 − t1, Δ) and effect sizes (Cohen’s *d_z_*) for all three groups.

	Hip			Knee			Ankle		
	t1	Δ	C*d_z_*	t1	Δ	C*d_z_*	t1	Δ	C*d_z_*
EQ-5D 5L mobility	2.12 ± 1.07	0.50 ± 1.19	0.15	1.86 ± 0.89	0.68 ± 0.92	0.02	1.90 ± 0.92	0.76 ± 0.82	0.10
EQ-5D 5L self-care	1.46 ± 0.84	0.03 ± 0.97	0.02	1.16 ± 0.45	−0.02 ± 0.44	0.05	1.05 ± 0.27	0.05 ± 0.27	0.12
EQ-5D 5L usual activities	2.01 ± 1.14	−0.14 ± 1.08	0.13	2.02 ± 1.01	−0.34 ± 0.93 **	0.36	1.75 ± 1.01	−0.21 ± 1.04	0.20
EQ-5D 5L pain/discomfort	2.34 ± 0.96	−0.11 ± 0.75	0.15	2.40 ± 0.68	−0.31 ± 0.70***	0.44	2.49 ± 0.76	−0.28 ± 0.75 **	0.36
EQ-5D 5L anxiety/depression	1.49 ± 0.79	−0.06 ± 0.91	0.07	1.29 ± 0.52	0.00 ± 0.53	0.00	1.34 ± 0.69	−0.04 ± 0.72	0.05
EQ-5D 5L health	63.85 ± 18.48	3.84 ± 25.52	0.15	64.69 ± 19.46	9.05 ± 20.53 ***	0.44	67.01 ± 17.76	6.91 ± 19.75 **	0.35
NPRS	3.51 ± 2.26	0.49 ± 2.09 *	0.24	3.45 ± 1.73	−0.83 ± 1.57 ***	0.53	3.65 ± 2.18	−0.96 ± 1.79 ***	0.54
HAQ-DI	0.53 ± 0.49	0.92 ± 0.33 *	0.27	0.30 ± 0.30	−0.05 ± 0.21 *	0.25	0.20 ± 0.24	−0.04 ± 0.17 *	0.24
WOMAC score	67.74 ± 46.05	−16.43 ± 31.65 ***	0.52	58.79 ± 37.15	−15.99 ± 28.68 ***	0.56	53.94 ± 40.91	−16.46 ± 30.35 ***	0.54
aROM %	64.03 ± 12.33	5.84 ± 6.49 ***	0.90	74.42 ± 14.76	5.87 ± 6.65 ***	0.88	25.03 ± 13.61	4.73 ± 4.46 ***	1.06
TUG	15.75 ± 9.42	−2.98 ± 4.11 ***	0.72	10.09 ± 4.43	−1.64 ± 1.84 ***	0.89	10.13 ± 6.64	−2.01 ± 4.89 **	0.41

Mean ± SD; Δ, Change (t2 − t1); * *p* < 0.05; ** *p* < 0.01; *** *p* < 0.001; C*d_z_*, Cohen’s *d_z_*, paired sample *t*-test, Holm–Bonferroni corrected.

**Table 3:** Spearman’s rank correlation coefficients between CROMs and PROMs at baseline (t1) and after intervention (t2).

rho	Hip		Knee		Ankle	
t1	aROM % t1	TUG t1	aROM % t1	TUG t1	aROM % t1	TUG t1
EQ-5D 5L mobility t1	−0.284 **	0.466 ***	−0.398 ***	0.311 **	−0.407 ***	0.393 ***
EQ-5D 5L self-care t1	−0.256 **	0.493 ***	−0.309 **	0.293 **	−0.154	0.187
EQ-5D 5L usual activities t1	−0.158	0.445 ***	−0.213 *	0.274 **	−0.211	0.139
EQ-5D 5L pain t1	−0.205	0.206 *	−0.249 *	0.170	−0.214	0.173
EQ-5D 5L anxiety/depression t1	−0.147	0.314 ***	−0.073	0.148	−0.112	0.161
EQ-5D 5L health t1	0.088	−0.205 *	0.289 **	−0.205 *	0.275 *	−0.340 **
NPRS t1	−0.248 **	0.197 *	−0.271 *	0.228 *	−0.315 **	0.334 **
HAQ-DI t1	−0.552 ***	0.713 ***	−0.531 ***	0.591 ***	−0.266 *	0.573 ***
WOMAC score t1	−0.407 ***	0.497 ***	−0.417 ***	0.447 ***	−0.345 **	0.388 ***
rMean [25]	0.262	0.394	0.307	0.298	0.257	0.295
rho	Hip		Knee		Ankle	
t2	aROM % t2	TUG t2	aROM % t2	TUG t2	aROM % t2	TUG t2
EQ-5D 5L mobility t2	−0.281 **	0.440 ***	−0.393 ***	0.396 ***	−0.362 **	0.439 ***
EQ-5D 5L self-care t2	−0.258 *	0.483 ***	−0.417 ***	0.206	−0.226	0.350 **
EQ-5D 5L usual activities t2	−0.284 **	0.518 ***	−0.255 *	0.368 **	−0.206	0.547 ***
EQ-5D 5L pain t2	−0.144	0.218 *	−0.215	0.147	−0.193	0.251 *
EQ-5D 5L anxiety/depression t2	−0.064	0.246 *	0.018	0.109	−0.195	0.202
EQ-5D 5L health t2	0.115	−0.228 *	0.407 ***	−0.272 *	0.139	−0.389 **
NPRS t2	−0.137	0.014	−0.314 **	0.160	−0.138	0.348 **
HAQ-DI t2	−0.426 ***	0.711 ***	−0.494 ***	0.606 ***	−0.262 *	0.597 ***
WOMAC score t2	−0.426 ***	0.477 ***	−0.452 ***	0.371 **	−0.219	0.481 ***
rMean [25]	0.238	0.373	0.331	0.294	0.217	0.403

rho, Spearman’s rank correlation; * *p* < 0.05; ** *p* < 0.01; *** *p* < 0.001.

**Table 4:** Consistency of performance between PROMs and CROMs for all three groups.

Hip	aROM % t2 + Δ			TUG t2 + Δ		
Consistency	High	Average	Low	High	Average	Low
EQ-5D 5L mobility t2 + Δ	65.6%	20.8%	13.5%	59.4%	29.2%	11.3%
EQ-5D 5L self-care t2 + Δ	59.4%	20.8%	19.8%	53.8%	31.1%	15.1%
EQ-5D 5L usual activities t2 + Δ	63.5%	26.0%	10.4%	64.2%	23.6%	12.3%
EQ-5D 5L pain t2 + Δ	60.4%	28.1%	11.5%	61.3%	24.5%	14.2%
EQ-5D 5L anxiety/depression t2 + Δ	52.1%	31.3%	16.7%	50.9%	34.9%	14.2%
EQ-5D 5L health t2 + Δ	52.1%	31.3%	16.7%	47.2%	35.8%	17.0%
NPRS t2 + Δ	49.0%	35.4%	15.6%	60.4%	23.6%	16.0%
HAQ t2 + Δ	56.3%	30.2%	13.5%	61.3%	27.4%	11.3%
WOMAC score t2 + Δ	52.1%	39.4%	8.5%	56.7%	30.8%	12.5%
Mean all scores	56.7%	29.3%	14.0%	57.2%	29.0%	13.8%
Knee	aROM % t2 + Δ			TUG t2 + Δ		
Consistency	high	average	low	high	average	low
EQ-5D 5L mobility t2 + Δ	62.3%	28.6%	9.1%	61.0%	30.5%	8.5%
EQ-5D 5L self-care t2 + Δ	67.5%	24.7%	7.8%	64.6%	29.3%	6.1%
EQ-5D 5L usual activities t2 + Δ	62.3%	27.3%	10.4%	56.1%	32.9%	11.0%
EQ-5D 5L pain t2 + Δ	57.1%	31.2%	11.7%	63.4%	24.4%	12.2%
EQ-5D 5L anxiety /depression t2 + Δ	53.2%	33.8%	13.0%	64.6%	23.2%	12.2%
EQ-5D 5L health t2 + Δ	63.2%	25.0%	11.8%	59.3%	27.2%	13.6%
NPRS t2 + Δ	59.7%	28.6%	11.7%	61.0%	28.0%	11.0%
HAQ-DI t2 + Δ	64.9%	23.4%	11.7%	65.9%	25.6%	8.5%
WOMAC score t2 + Δ	61.0%	24.7%	14.3%	59.8%	26.8%	13.4%
Mean all scores	61.3%	27.5%	11.3%	61.7%	27.5%	10.7%
Ankle	aROM % t2 + Δ			TUG t2 + Δ		
Consistency	high	average	low	high	average	low
EQ-5D 5L mobility t2 + Δ	63.2%	30.9%	5.9%	64.9%	29.7%	5.4%
EQ-5D 5L self-care t2 + Δ	64.7%	32.4%	2.9%	67.6%	31.1%	1.4%
EQ-5D 5L usual activities t2 + Δ	55.9%	30.9%	13.2%	70.3%	20.3%	9.5%
EQ-5D 5L pain t2 + Δ	45.6%	44.1%	10.3%	60.8%	25.7%	13.5%
EQ-5D 5L anxiety/depression t2 + Δ	60.3%	26.5%	13.2%	52.7%	37.8%	9.5%
EQ-5D 5L health t2 + Δ	58.8%	25.0%	16.2%	70.3%	18.9%	10.8%
NPRS t2 + Δ	47.1%	39.7%	13.2%	64.9%	23.0%	12.2%
HAQ-DI t2 + Δ	64.7%	30.9%	4.4%	60.8%	33.8%	5.4%
WOMAC score t2 + Δ	50.0%	38.2%	11.8%	64.9%	23.0%	12.2%
Mean all scores	56.7%	33.2%	10.1%	64.1%	27.0%	8.9%

Consistency of performance (t2 + Δ interval centred percentile rank standard norm equivalents/normal scores using Rankit’s Formula): High 0–1 SD, Average > 1SD–2SD, Low > 2SD.

## Data Availability

The datasets analysed in this manuscript are not publicly available, because of ethical and legal restrictions (data contain potentially identifying and sensitive patient information). If not already reported within this work, the authors may provide descriptive data on individual medical indicators for admission and discharge or the expected change due to inpatient health care for various groups and diagnoses. Requests for access to anonymised datasets should be directed to the corresponding author.
